# Gut Microbiota in Primary Osteoporosis: a Systematic Review

**DOI:** 10.1007/s43657-024-00164-y

**Published:** 2024-07-12

**Authors:** Jiangxun Ji, Feihong Cai, Chunchun Yuan, Chen Huang, Haitao Zhang, Chuanglong Xu, Wendong Suo, Wenhao Zhu, Binhao Shi, Dezhi Tang, Yongjun Wang

**Affiliations:** 1 Longhua Hospital, Shanghai University of Traditional Chinese Medicine, Shanghai, 200032 China; 2 Key Laboratory of Theory and Therapy of Muscles and Bones, Ministry of Education, Shanghai, 200032 China; 3 Spine Institute, Shanghai Academy of Traditional Chinese Medicine, Shanghai, 200032 China; 4 Ningxia Traditional Chinese Medicine Hospital and Chinese Medicine Research Center, Yinchuan, 750021 China; 5 Shanghai University of Traditional Chinese Medicine, Shanghai, 201203 China; 6 Shanghai Municipal Hospital of Traditional Chinese Medicine, Shanghai University of Traditional Chinese Medicine, Shanghai, 200071 China

**Keywords:** Bone Mineral Status, Osteoporosis, Gut Microbiota, Metagenomes

## Abstract

The link between gut microbiota (GM) and primary osteoporosis has garnered substantial attention. In this study, we conducted a systematic review encompassing 10 studies that employed high-throughput sequencing methodologies. Notably, the taxonomic profiles exhibited pronounced heterogeneity across reviewed studies. A combined analysis of mechanistical studies screened out potential targets for osteoporosis prevention and treatment. We appraised the limitations in previous studies, and proposed suggestions for optimizing sequencing techniques and experimental designs. Larger-size longitudinal cohorts based on multi-omics and extended phenotypes are recommended to comprehensively characterize the involvement of GM in the pathogenesis of osteoporosis.

**Supplementary Information:**

The online version contains supplementary material available at 10.1007/s43657-024-00164-y.

In 2012, Sjögren et al. (2012) found that colonization of the germ-free mice with gut microbiota (GM) from normal mice normalized the increased bone mass density (BMD) in germ-free mice, providing compelling evidence that GM links with bone metabolism. More studies further illustrated mechanisms and pathways possibly involved in this link, including the gut-brain axis, gut-liver axis, and immune system regulation (Supplementary Part and Fig. 1). Thus, GM may serve as a diagnostic or therapeutical biomarker for osteoporosis.

**Fig. 1:**
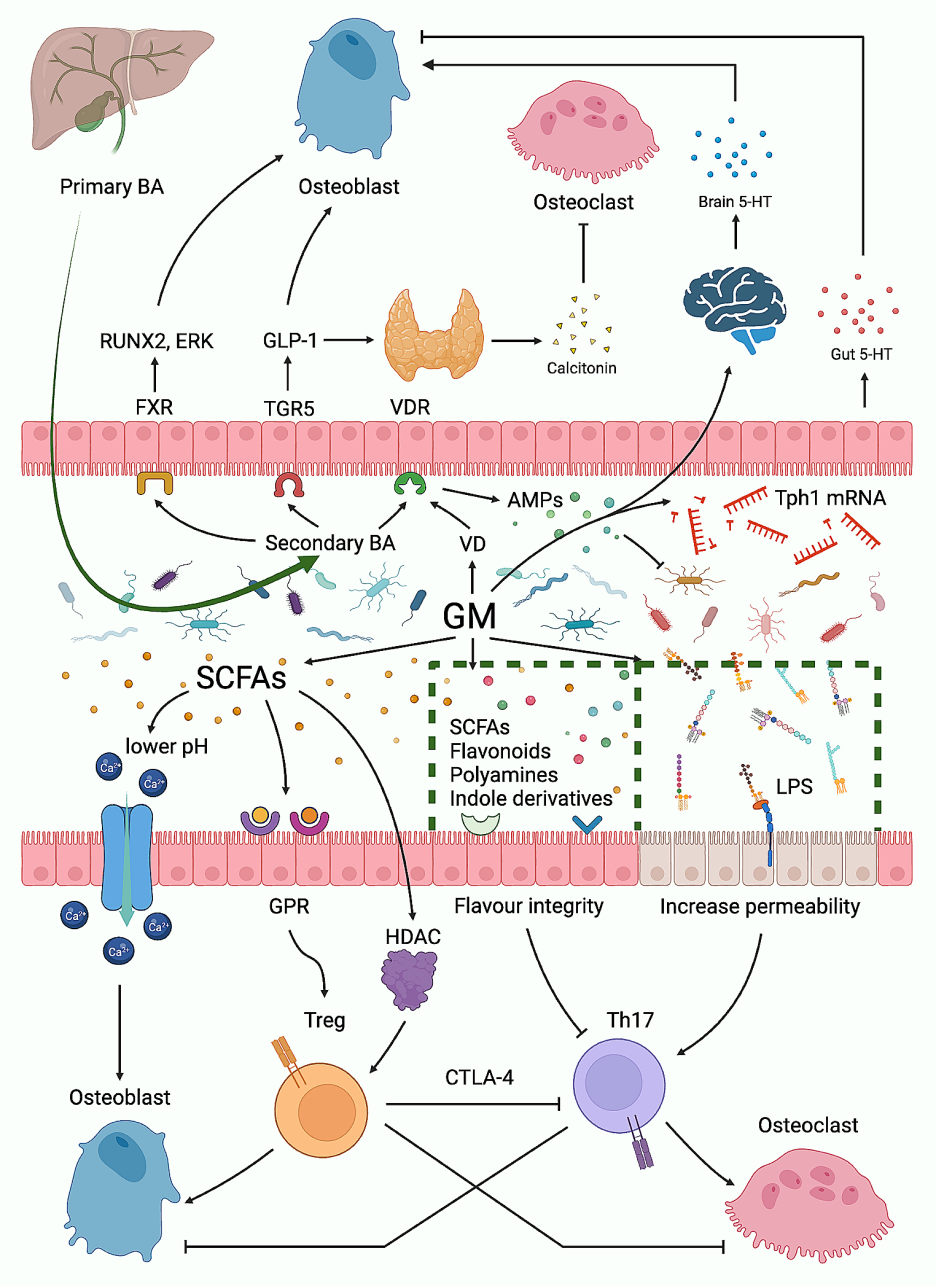
Pathways GM influences bone metabolism The gut microbiota influences bone metabolism through three main pathways: first, by converting primary bile acids into secondary bile acids, thereby activating a series of downstream pathways; second, by regulating the secretion of 5-HT; and third, by secreting substances such as short-chain fatty acids that affect the balance between Tregs and Th17. BA = Bile acid; FXR = Farnesoid X receptor; GLP-1 = Glucagon-like peptide-1; TGR5 = Takeda G protein-coupled receptor 5; VDR = Vitamin D receptor; 5-HT = 5-hydroxytryptamine; AMP = Antimicrobial peptide; VD = Vitamin D; SCFAs = Short-chain fatty acids; GM = Gut microbiota; LPS = Lipopolysaccharide; GPR = G protein-coupled receptor; HDAC = Histone deacetylase; CTLA-4 = Cytotoxic T lymphocyte-associated protein 4

Microbiota-host studies often start with finding microbial structure alterations, and their associations with various phenotypes. Followed by the employment of animal models to explore molecular mechanisms. Here we systematically reviewed previous observational outcomes, hoping to provide directions for future research.

## Main Finding: High Heterogeneity in Previous Articles

**Fig. 2:**
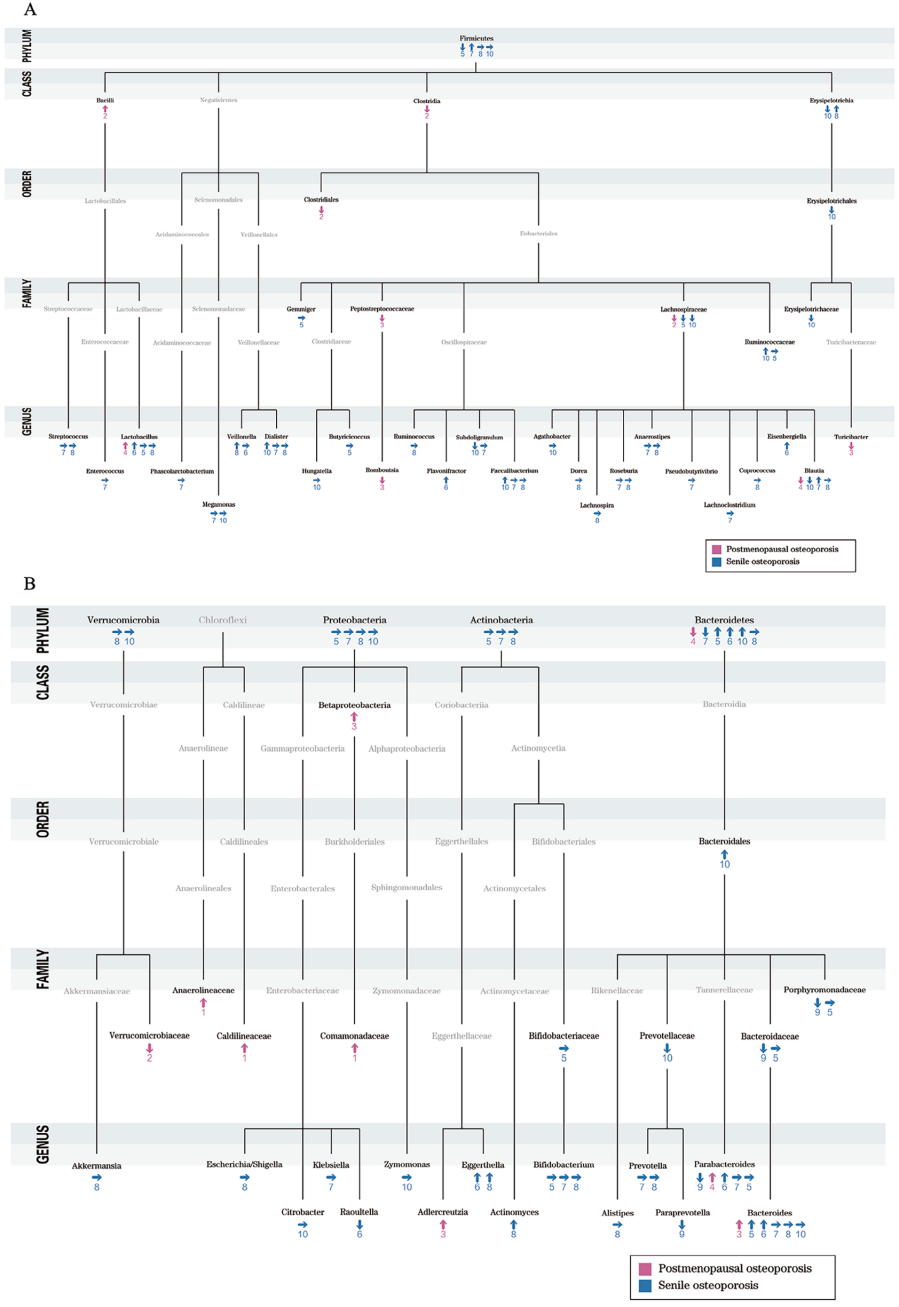
Taxonomic findings in primary osteoporosis Taxonomic differences observed in at least two of the reviewed studies at the phylum, order, family and genus levels. Note: in the context of these associations, “↑” indicates a higher relative abundance in osteoporosis group, “↓” signifies a lower relative abundance in osteoporosis group, and “→” denotes no significant relative abundance change. Studies: (1) (Wang et al. 2017a); (2) (Lv et al. 2021); (3) (Rettedal et al. 2021); (4) (He et al. 2020); (5) (Di et al. 2021); (6) (Wei et al. 2021); (7) (Wang et al. 2017b); (8) (Das et al. 2019); (9) (Qin et al. 2021); (10) (Xu et al. 2020) (All studies are referenced in Supplementary Part).

Following a systematic analysis of included articles (Fig. S1 and Table S1-4), we found that available evidence does not support a significant difference in alpha diversity (Table S5), but in beta diversity between individuals with primary osteoporosis and their healthy counterparts (Table S6), and taxonomic analysis results exhibit a high variability across studies (Fig. 2 and Table S7).

## Sources of Heterogeneity

The heterogeneity across studies may originate from sequencing methods. 16S rRNA sequencing, as a type of amplicon sequencing, only sequences hypervariable regions (V1-V9) of the bacterial 16S rRNA gene. Therefore, its results differ from those of whole genome sequencing.

Another source may be protocol-related, mainly pertaining to sampling and storage procedures, DNA extraction techniques, primers, sequencing platforms, data processing (including pipelines) and reference databases.

High inter-individual variability of GM, combined with small sample size, inadequate clinical data, and failure to control variables, all undermine the reproductivity of research results. The variability of GM across individuals is comparable to that across different environments, as seen in the Earth Microbiome Project (Allaband et al. 2019). Moreover, the small sample sizes of included studies discounted the statistical power to address the relationship between GM and BMD. In addition, only some of the studies collected clinical data, controlled variables, and excluded confounders by large. Finally, GM always shows a dynamic profile. Human Microbiome Project showed that 16% of subjects experienced a change in their GM enterotype over a 6-month period (Costea et al. 2018).

## Orientation of Future Research

Firstly, studies on GM should strictly adhere to guidelines. Researchers at the Microbiome Quality Control Project discovered that variations in DNA extraction, amplification protocols, and raw signal analysis may rival those in GM between individuals (Sinha et al. 2017). Given this, a group of clinicians, epidemiologists, biologists, bioinformaticians, and statisticians developed the Strengthening the Organizing and Reporting of Microbiome Studies (STORMS) checklist in 2020.

Sequencing method and experimental design decide the success or failure of a study. Only two studies employed metagenomic sequencing, and the remainder opted for 16S rRNA sequencing, which harbors inherent limitations. One such limitation is related to the different affinities of primers to the sequences. Additionally, 16S rRNA sequencing lacks taxonomic resolution at species and strain levels. Those species and strains may be genomically close, but functionally distinct, either probiotic or pathogenic. GM contains bacteria, archaea, fungi, eukaryotes, viruses, and phages. However, amplicon sequencing cannot detect genes from eukaryotes, viruses, and archaea. In contrast, shotgun metagenomics retrieves all genomes from the microbial community, providing insights into microbiome functions via database references. This tool offers a possibility for subsequent studies linking human phenotypes to GM. Besides, new sequencing models and algorithms are emerging to sequence low-abundance species in GM (Jin et al. 2022). Moreover, strain-level GM variation is also implicated in disease pathogenesis, for that the strains of one species may be functionally different. Researchers are encouraged to explore new technologies, like microbe-seq (Zheng et al. 2022).

As with experimental design, a large longitudinal prospective cohort study is expected to be established. Previous cross-sectional studies have identified differences in the GM of osteoporosis patients compared to controls at a specific time, but inconsistencies in findings have made it difficult to establish a causal relationship between GM dysbiosis and osteoporosis. Those inconsistencies may derive from confounders, such as diet, glucocorticoid administration, and age, which are also risk factors for osteoporosis. A well-designed longitudinal study with extensive clinical data and statistical methods, such as Mendelian randomization, may address this issue and provide targets for subsequent in vivo animal studies. New technology has allowed to construct a flora of 104 bacterial species in vitro and its implantation into germ-free mice (Cheng et al. 2022), which greatly enhances the credibility of animal validation models. In addition, a large longitudinal study has verified the value of microbiota changes in clinical diagnoses, as seen by the changes in GM composition before disease progression in patients with colorectal cancer (Chong et al. 2022).

Integrating multi-omics data should be strengthened. In the gut, various microorganisms perform similar functions, and their dysfunctions may be compensated by others. Given their complex interactions, such as cooperation and competition, it is hard to understand, utilize, and regulate them for preventing and treating diseases. However, these interactions are close and overlapped, endowing different diseases to similar GM profiles. Maintaining normal GM can potentially benefit the overall health of the host. New therapeutic modalities have been developed by exploiting the complex relationships between microbes. For example, researchers have used phages to precisely target *Klebsiella pneumoniae*, an intestinal bacterium associated with inflammatory bowel disease, thereby significantly reducing the intestinal inflammation and tissue damage caused by this strain (Federici et al. 2022). The complex relationship between microorganisms presents both challenges and opportunities. To elucidate the complex inter-microbial relationships, ecological measures and multi-omics approaches (e.g., genomics, metatranscriptomics, metabolomics, proteomics, immunomics, lipidomics) can provide insights into the molecular pathways regulating the gut-bone axis. Additionally, new techniques, like Record-seq, can record gene expression shifts of targeted microorganisms facing a new niche, including introducing a new microorganism, thus aiming to elucidate microbial interactions.

Emerging evidence indicates that microbiota residing in various mucosal sites collectively influence host bone metabolism. Kitamoto et al. (2020) have discovered significant rises in *Klebsiella* and *Aspergillus* in the oral mucosa of periodontitis mice. These oral bacteria also colonized the lower digestive tract and activated macrophage inflammasomes in the gut. Through experiments, they found that pathobiont-specific T-helper 17 memory cells could migrate from the oral cavity to the intestine through lymphatic vessels. In the gut, when encountering *Klebsiella* and *Enterobacter* from the oral cavity, these memory cells quickly became activated, promoting intestinal inflammation. These findings confirm an interaction between oral microbiota and GM. In bone metabolism, oral microbiota and GM may collaborate to regulate the activity of osteoblasts and osteoclasts by tilting the balance between T-helper 17 cells and regulatory T cells. The concept of a “common mucosal immunological system” was initially introduced five decades ago, when structural similarities were observed between the lymphoid tissue associated with the bronchi and that located in the gastrointestinal tract. In recent years, it has been recognized that except for structural similarity, tissue of various mucosal sites also plays a synergistic role in the regulation of the immune system. Thus, future research on osteoporosis should spare efforts to uncover the interactions between microbiota at multiple sites.

GM may be exploited to realize early detection, diagnosis, staging and treatment of osteoporosis. The association of GM in osteoporosis should be further explored using studies based on new sequencing tools, multi-omics data, established protocols, various phenotypes, and large cohorts. A comprehensive understanding of GM’s role in osteoporosis is expected to bring forth revolutionary treatments.

## Electronic Supplementary Material

Below is the link to the electronic supplementary material.

Supplementary Material 1

Supplementary Material 2

## Data Availability

Data sharing not applicable to this article as no datasets were generated or analyzed during the current study.

## Abbreviations

BMD: Bone mineral density.
GM: Gut microbiota
STORMS: Strengthening the Organizing and Reporting of Microbiome Studies
