# Supplementary material for: Gut Microbiota in Primary Osteoporosis: a Systematic Review
Source: Phenomics. 2024 Jul 12;4(3):293–7. doi: 10.1007/s43657-024-00164-y (PMC11467150; doi:10.1007/s43657-024-00164-y)
Supplement: Supplementary file 1 — Supplementary Material 1 [file 43657_2024_164_MOESM1_ESM.docx]

^19th^ May, 2023

Respected Editors,

In the field of orthopaedics, osteoporosis has always been a hot topic, and its incidence is increasing, while the treatment methods are lingering behind with no major breakthrough for years. However, based on gut microbiota, it is promising to early detect, diagnose, and stage osteoporosis, and to prevent and treat osteoporosis by regulating intestinal flora. Such a concept is already being practiced in inflammatory bowel disease and bowel cancer.

Current animal studies have confirmed that dysbiosis in the gut flora play a key role in the development of osteoporosis, and animal studies have identified three core mechanisms: the brain-gut axis, the biliary cycle, and gut flora metabolite - human immune system interactions. The evidence that gut flora can influence bone metabolism is clear, but a causal relationship between osteoporosis and gut flora dysbiosis has not been established despite years of studying.

The establishment of such a causal relationship, and the future clinical application of gut microbiota therapy or modulation in the field of osteoporosis, can only be flower in the mirror, moon on the water without solid clinical supports. Based on the PRISMA principle, we have systematically screened all clinical studies in this field and found that the small sample size and heterogeneity of the existing studies, as well as the shortcomings of the experimental protocols, have made it difficult to achieve a major breakthrough in this field. In this regard, this review not only systematically reviews the existing clinical studies and outlines the main mechanisms in which gut microbiota affects bone metabolism, but also extensively reviews the literature in other fields to propose corresponding solutions, which includes: 1) the development and exploration of new sequencing methods to increase the depth and breadth of sequencing; 2) the optimisation of experimental sequencing and bioinformatic analysis by reference and comparison of experimental protocols; 3) the comprehensive collection of subjects’ phenotypes; 4) the establishment of large prospective longitudinal cohorts that use multi-omics analysis and integrate environmental and personal factors.

This study was supported by the National Key R&D Program of China (2018YFC1704300), the National Natural Science Foundation of China (81730107, 11871456, 81973883), the Shanghai Municipal Science and Technology Major Project (2017SHZDZX01), the Program for Innovative Research Team of Ministry of Education of China (IRT1270), the Program for Innovative Research Team of Ministry of Science and Technology of China (2015RA4002), and the Inheritance and Innovation Team Project of National Traditional Chinese Medicine (ZYYCXTD-C-202202).

We declare that this manuscript is approved for publication by each author without conflict of interest. We confirm that the material is original and that it has been neither published elsewhere nor submitted for publication simultaneously. In addition, we declare that if this article is accepted, it will not be published elsewhere in the same form, in English or any other language, without the written consent of the copyright holder. All authors have approved the manuscript and agreed with submission to Ageing Research Reviews*.*

We hope this paper is suitable for “ Ageing Research Reviews ”.

We really appreciate your consideration of our manuscript, and look forward to receiving comments from the reviewers.

Yours sincerely,

Dr. Yongjun Wang

M.D. & PhD., Professor, Chief Physician

Vice President, Shanghai University of Traditional Chinese Medicine

Vice President, Shanghai Academy of Traditional Chinese Medicine

Address: 1200 Cailun Road, Pudong, Shanghai, 201203, China.P.R

Tel: +86（021）64385700

E-mail: [yjwang8888@126.com](mailto:yjwang8888@126.com)
