# Supplementary material for: Gut Microbiota in Primary Osteoporosis: a Systematic Review
Source: Phenomics. 2024 Jul 12;4(3):293–7. doi: 10.1007/s43657-024-00164-y (PMC11467150; doi:10.1007/s43657-024-00164-y)
Supplement: Supplementary file 2 — Supplementary Material 2 [file 43657_2024_164_MOESM2_ESM.docx]

**Supplementary Part for**

Gut microbiota in primary osteoporosis: a systematic review

Jiangxun Ji^a,b,c^*, Feihong Cai^a,b,c^*, Chunchun Yuan^a,b,c^*, Chen Huang^a,b,c^, Haitao Zhang^a,b,c^, Chuanglong Xu^d^, Wendong Suo^a,b,c^, Wenhao Zhu^a^, Binhao Shi^a,b,c#^, Dezhi Tang^a,b,c#^, Yongjun Wang^a,b,c,e#^

^a^ Longhua Hospital, Shanghai University of Traditional Chinese Medicine, Shanghai, 200032, China

^b^ Key Laboratory of Theory and Therapy of Muscles and Bones, Ministry of Education, Shanghai, 200032, China

^c^ Spine Institute, Shanghai Academy of Traditional Chinese Medicine, Shanghai, 200032, China

^d^ Ningxia Traditional Chinese Medicine Hospital and Chinese Medicine Research Center, Yinchuan, 750021, China

^e^ Shanghai University of Traditional Chinese Medicine, Shanghai, 201203, China

* Contributed equally to this work.

^#^Correspondence: Yongjun Wang (yjwang@shutcm.edu.cn) or Dezhi Tang ([dztang@shutcm.edu.cn）or](mailto:dztang@shutcm.edu.cn）or) Binhao Shi (s4512shibinhao@163.com)

**Abbreviations**

1,25-(OH)2D3 1,25-dihydroxy vitamin D3

5-HT 5-hydroxytryptamine

7-DHC 7-dehydrocholesterol

BMD Bone mineral density

CTLA-4 Cytotoxic T lymphocyte-associated protein 4

VDR Vitamin D receptor

FXR Farnesoid X receptor

GLP-1 Glucagon-like peptide-1

GM Gut microbiota

HDAC Histone deacetylase

IGF-1 Insulin-like growth factor 1

LPS Lipopolysaccharides

NF-κB Nuclear factor kappa-light-chain-enhancer of activated B cells

RANKL Receptor activator of nuclear factor kappa-Β ligand

SCF Soluble corn fibre

SCFAs Short-chain fatty acids

TGF-β Transforming growth factor-β

TGR5 Takeda G protein–coupled receptor 5

TH17 T-helper 17 cells

TLR2 Toll-like receptor 2

TNF-α Tumor necrosis factor alpha

Tregs Regulatory T cells

OTUs Operational taxonomic units

PTH Parathyroid hormone

**Materials and Methods**

Search strategy

To ensure a rigorous and systematic review process, we followed the PRISMA 2020 guidelines to formulate database retrieval strategies (Page et al. 2021). Two researchers (JJX and CFH) independently conducted a comprehensive literature search using major English databases, including PubMed, Cochrane Library, Embase, and Web of Science, as well as Chinese databases, including China National Knowledge Infrastructure Database, Sinomed, China Science and Technology Journal Database, and Wanfang Database. We also manually searched for relevant articles and clinical studies to obtain as much literature as possible. No restrictions were placed on language or publication status for this review. For a detailed description of our search strategy, please refer to Supplementary Search Strategy.

Selection criteria of the studies

Inclusion criteria

Included studies should be clinical trials targeted to adults older than 50 or postmenopausal women with natural menopause, using high-throughput sequencing methods to quantify microorganisms. Furthermore, all the studies should report both GM diversity and bone health parameters.

Exclusion criteria

Review papers, meta-analyses, abstracts or conference proceedings, articles with duplicate data, case reports; Studies exclusively using animal models or other non-GM biomarkers (e.g., saliva, blood, skin).

Study selection and data extraction

Two researchers (JJX and CFH) independently screened the studies based on selection criteria, primarily through the title, abstract, and keywords for preliminary screening. Then, they reread the full text for re-screening and cross-checked the results. Divergences were resolved by a senior researcher (YCC). The contents extracted were: first author, year of publication, country, age range, gender, sample size, sequencing methods, and outcomes.

Assessment of risk of bias

A new “risk of bias assessment” tool developed by the National Heart, Lung and Blood Institute was used to evaluate the quality of the included literature. The risk of bias tool contains fourteen questions. Each item was assessed to be “Yes,” “No,” “Cannot determine,” or “Not applicable.”

**Results**

Study selection and characteristic

We retrieved 1741 articles through the databases. After the strict screening (**Fig.** S1), we included ten articles. Most of the studies are cross-sectional and conducted in different parts of China as showed in **Table** S1.

Confounding variables control across reviewed literature

The included literature more or less controlled for confounding factors that might affect GM and(or) bone mass density (BMD). Here we selected several well-established and well-studied confounding factors that undoubtedly influence GM, which include age (Yatsunenko et al. 2012), body mass index (BMI) (Aoun et al. 2020), gender (Alswat 2017), bowel diseases (Nishida et al. 2018), antibiotics (Lange et al. 2016), bone metabolism influencing drugs (Pushalkar et al. 2014), prebiotics (McCabe et al. 2015), probiotics (McCabe et al. 2015), smoking (Li et al. 2020a) and alcohol consumption (Engen et al. 2015) to examine whether the included literature effectively eliminated the influence of confounding factors (**Table** S2). Only two literature (Das et al. 2019; Wei et al. 2021) strictly controlled the effect of confounding factors.

Methodological summary

Of the ten studies included in our systematic review, two utilized shotgun metagenomic sequencing, while the remaining eight used 16s rRNA sequencing with Illumina platform, except for one (Xu et al. 2020). All studies choose V3-V4 to sequence and use operational taxonomic units (OTUs) as a clustering approach. However, the specific methods used for quality control, pipeline, and reference databases varied across the studies (**Table** S3).

Quality assessment

According to the quality assessment tool, we classified two literature as good quality (Das et al. 2019; Wei et al. 2021), five (Di et al. 2021; He et al. 2020; Qin et al. 2021; Rettedal et al. 2021; Xu et al. 2020) as fair quality, and three (Lv et al. 2021; Wang et al. 2017a; Wang et al. 2017b) as poor quality. The studies with a poor-quality rating had limitations such as a small sample size, insufficient control of confounding variables, and less robust data processing methods. A more detailed breakdown of the quality rating for each study can be found in **Table** S4.

GM and primary osteoporosis

Alpha diversity

Articles examining GM and primary osteoporosis reported alpha diversity with varying results (**Table** S5).

Beta diversity

Eight of the nine articles investigating changes in the GM of primary osteoporosis patients reported beta diversity and found that the osteoporosis group distinguished from the control group, except Qin et al. (2021) (**Table** S6).

Taxonomic findings

Given the limited number of studies examining the GM differences between primary osteoporotic patients and healthy individuals, though many taxonomic differences were uncovered, there was little replication between studies, and often, findings did not corroborate between studies (**Table** S7 and **Fig.** 2).

**Discussion**

**Interpretation of findings of alpha diversity**

Alpha diversity has long been used as a measure of GM health, with higher diversity assumed to indicate better health. However, the relationship between alpha diversity and individual health is complex and not fully understood (Reese and Dunn 2018). For instance, a high alpha diversity does not necessarily indicate healthy functioning or stability in response to disturbance. Moreover, experimental techniques and data processing methods can introduce biases in the range of alpha diversity indices. Accordingly, recent studies have found a weak or non-existent association between alpha diversity and individual health. For example, investigations of GM in vertebrates across consecutive breeding seasons found no correlation between alpha diversity and individual health (Worsley et al. 2021). Similarly, studies in humans with Parkinson’s disease or multiple sclerosis have demonstrated no significant differences in alpha diversity compared to healthy controls (Plassais et al. 2021). In accordance with all these findings, the articles included in this review also show that there is not sufficient evidence to support a difference in alpha diversity between the osteoporotic group and healthy controls.

**Brief review of mechanisms GM influences bone metabolism**

In contrast to alpha diversity, this review reveals that individuals with primary osteoporosis exhibit distinct GM composition when compared to healthy controls. This clinical observation is supported and explained by numerous potential pathways through which GM influences bone metabolism (**Fig.** 1).

GM can produce various metabolites that affect the host’s immune system and help maintain gut barrier integrity, which, in turn, can influence the absorption of ions and vitamins involved in bone metabolism. For example, GM-derived metabolites like polyamines, short-chain fatty acids (SCFAs), flavonoids, and indole derivatives can help maintain gut barrier integrity (Keirns et al. 2020). However, lipopolysaccharides (LPS) can increase intestinal wall permeability and enter the bloodstream to promote T-helper 17 cells (Th17) differentiation. As a result, it increases host inflammation levels and intestinal wall permeability (Guo et al. 2013). By inhibiting histone deacetylase (HDAC) activity and increasing GPR41, GPR43, and GPR109A activity, SCFAs can promote regulatory T cells (Tregs) production (Arpaia et al. 2013; Davie 2003; Lucas et al. 2018; Smith et al. 2013). Tregs, anti-inflammatory cells suppressing and regulating the activity of inflammatory T cells, release Wnt10b protein to promote osteoblast genesis and secrete IL-10, transforming growth factor-β (TGF-β), and IL-4 to inhibit osteoclasts (Luo et al. 2011; Taylor et al. 2006; Tyagi et al. 2018). Tregs also inhibit Th17 differentiation via cytotoxic T lymphocyte-associated protein 4 (CTLA-4) (Kong et al. 1999; Lucas et al. 2018). Th17, unlike Tregs, increases osteoclast activity by releasing IL-17, TNF-α, and IL-6 (Sapra et al. 2021). Furthermore, SCFAs such as propionate and butyrate downregulate essential osteoclast genes such as TRAF6 and NFATc1 by enhancing glycolysis (Lucas et al. 2018). Interestingly, studies have found that parathyroid hormone (PTH), a crucial regulator of skeletal development, cannot exert its anabolic role on bone mass in GM-depleted mice. However, re-establishing physiological levels of butyrate can reverse this trend (Li et al. 2020c). Additionally, SCFAs can lower intestinal pH levels and promote calcium absorption (Whisner et al. 2016).

Second, GM influences bone metabolism by regulating liver-related processes. Under the action of the GM, primary bile acid produced by the liver is converted into secondary bile acid. Secondary bile acid binds with farnesoid X receptor (FXR) and Takeda G protein–coupled receptor 5 (TGR5; also known as GPBAR1) on the intestinal wall and activates a series of signalling pathways to promote osteogenic activity (Cho et al. 2013), including stimulating RUNX2-mediated osteoblastic differentiation of bone marrow stromal cells (Boufker et al. 2011), promoting Glucagon-like peptide-1 (GLP-1) secretion which activates Wnt pathways (Liu and Habener 2008). GLP-1 also acts on the thyroid gland to promote the release of calcitonin, inhibiting osteoclast activity (Crespel et al. 1996). At the same time, by producing SCFAs, the GM may increase liver and adipose tissue insulin-like growth factor 1 (IGF-1) production, which promotes osteosynthesis (Koh et al. 2016; Yan et al. 2016). In addition, a randomized controlled trial of 127 otherwise healthy hypercholesterolemic adults shows that oral supplement of *L. reuteri NCIMB 30242* increases circulating 25-hydroxyvitamin D by promoting hepatic 25-hydroxylase activity or 7-dehydrocholesterol (7-DHC) concentration (Jones et al. 2013). Vitamin D is essential for regulating calcium metabolism by increasing intestinal calcium absorption and calcium bone resorption (Laird et al. 2010), and activating vitamin D receptor (VDR) in the gut. The importance of VDR’s role in skeletal health has been emerging since studies found that activating VDR favors the integrity of the gut barrier and triggers antimicrobial peptides (AMP) production to defend against pathogenic microbiome (Akimbekov et al. 2020). A study found that 1,25-dihydroxy vitamin D3 (1,25-(OH)2D3) attenuated dextran sodium sulfate induced paracellular permeability by increasing the expression of the tight junction proteins (zo-1, occludin, and claudin-1) in the mucosa. Secondary bile acids are also regulators of VDR in the gut (Makishima et al. 2002; Wang et al. 2016), and *Parabacteroides* are believed to contain pathways involved in secondary bile acid metabolism according to a genome-wide association involving 1812 individuals (Wang et al. 2016).

Third, GM affects bone metabolism through the brain-gut-bone axis by modulating the 5-hydroxytryptamine (5-HT) signal transduction system. GM upregulates the expression of Tph1 mRNA, which acts on enterochromaffin cells and promotes the release of intestinal 5-HT. Intestinal 5-HT has an inhibitory effect on osteoblast activity (Reigstad et al. 2015). In contrast, brain-derived 5-HT has an osteogenic effect, and GM influences the production of brain 5-HT through its metabolite leptin (Yadav et al. 2009).

**Interpretation of taxonomical differences**

GM comprises about 1200 bacterial species, which is varied personally. Despite this variation, four main phyla - *Firmicutes*, *Bacteroides*, *Proteobacteria*, and *Actinobacteria* - represent 98% of GM (Ley et al. 2006). Two studies included in this review, Wang et al. (2017b) and Xu et al. (2020) found that the ratio of *Firmicutes*/*Bacteroidetes* was inversely related to BMD, a finding consistent with subsequent clinical trials (Cheng et al. 2022). However, animal studies did not show the same pattern. In fact, Yuan et al. (2022) found that the mean *Firmicutes*/*Bacteroidetes* ratio was significantly reduced in ovariectomized mice. Thus, further investigation is needed to better understand these discrepancies.

***Phylum Firmicutes***

Despite the known role of *Phylum Firmicutes* in promoting the fermentation of soluble corn fibre (SCF) and its positive impact on calcium absorption (D'Amelio and Sassi 2018), existing studies did not yield adequate evidence to support a difference in the abundance of *Phylum Firmicutes* between the GM of osteoporotic patients and healthy individuals. Several bacterial groups within the *Phylum Firmicutes* have demonstrated osteoprotective effects and have even been shown to facilitate bone healing in elderly individuals following fractures (Lei et al. 2016).

Commensal *Clostridia* supports the integrity of the gut barrier by releasing butyrate and promoting the differentiation of Tregs (Lopetuso et al. 2013). In studies conducted on adult mice, it was observed that oral administration of *Clostridia* strains led to a reduction in colitis and allergic diarrhea by stimulating the differentiation of Tregs. Within the *Clostridia* group, specific taxa such as *f_Lachnospiraceae* (*Clostridium XlVa*), *g_Blautia*, and *g_Faecalibacterium* have been identified as producers of SCFAs, which contribute to the overall health of the host (Belzer et al. 2017; Ríos-Covián et al. 2016). However, among the literature examined in this study, only *f_Lachnospiraceae* (*Clostridium XlVa*) exhibited significant differences between healthy individuals and patients with osteoporosis.

The abundance of *g_Dialister* is linked to BMD. Interestingly, certain bacteria within this group are capable of producing propionate via the succinate pathway, thereby promoting BMD (Koh et al. 2016). However, an increase in the abundance of *Dialister* is closely associated with elevated levels of IL-6, which can contribute to bone loss (Martínez et al. 2013).

*Veillonella*, a genus belonging to the *Firmicutes*, can metabolize isoflavone diadzin into equol, which is an estrogen analogue known for its potential osteoprotective properties (Jin et al. 2015).

The genus *Lactobacillus*, classified under *Firmicutes*, is widely recognized as a probiotic with numerous researches conducted on its subordinate strains. These bacteria have been found to influence human bone metabolism through various mechanisms. Firstly, different species within the *Lactobacillus* can modulate immunity, thereby impacting human bone metabolism. For instance, *Lactobacillus reuteri* has been shown to restore inosine levels and modify the disrupted metabolomic profile caused by Tregs deficiency, highlighting its immunomodulatory function and potential osteoprotective value (Cervantes-Barragan et al. 2017). Furthermore, *Lactobacillus reuteri* can induce the conversion of gut intraepithelial CD4+ T cells into Tregs by generating indole derivatives of tryptophan (Cervantes-Barragan et al. 2017). In a study involving 70 older women with low BMD, Nilsson et al. (2018) discovered that daily supplementation with *L. reuteri 6475* for 12 months resulted in reduced BMD loss. However, unlike the in vitro study, the in vivo study did not observe any significant effects of *L. reuteri 6475* supplementations on the tumor necrosis factor alpha (TNF-α) mediated immune response (Nilsson et al. 2018). Moreover, *Lactobacillus plantarum NK3* and *Bifidobacterium longum NK49* have shown potential in alleviating osteoporosis by suppressing nuclear factor kappa-light-chain-enhancer of activated B cells (NF-κB) linked TNF-α expression (Kim et al. 2019).

Species within the *Lactobacillus* genus affects calcium absorption in humans. Specifically, oral administration of *Lactobacillus reuteri* effectively increases serum levels of 1,25-(OH)2D3, which is crucial for calcium absorption (Jones et al. 2013). Similarly, in postmenopausal women, consumption of milk fermented with *Lactobacillus helveticus* has been demonstrated to enhance calcium absorption levels, benefiting bone health (Narva et al. 2004).

Various species within the *Lactobacillus* regulate bone metabolism by influencing the human endocrine system. Rat models under different nutritional statuses and physical activity have shown a positive correlation between the abundance of *Lactobacillus* and serum leptin levels (Queipo-Ortuno et al. 2013). Furthermore, oral consumption of dairy products containing *Lactobacillus plantarum* and *Lactobacillus acidophilus* has been demonstrated to reduce serum homocysteine levels by modulating the homocysteine metabolic pathway (Bhardwaj et al. 2022). In cases of osteoporosis resulting from estrogen deficiency, GM dysbiosis plays a significant role in the disease development. Germ-free mice, compared to normal mice, did not observe bone loss caused by sex hormone deficiency (Li et al. 2016). And treatment with probiotics such as *Lactobacillus rhamnosus GG* restored barrier integrity in steroid deficient mice (Li et al. 2016). While the beneficial effects of *Lactobacillus* have been interpreted from various perspectives, two of the included papers found a significantly higher relative abundance of *Lactobacillus* in the osteoporosis group (He et al. 2020; Wei et al. 2021).

***Phylum Bacteroidetes***

SCF promotes calcium absorption, and a high intake of SCF impacts the structure of GM. Specifically, it leads to an increase in the number of *Bacteroidetes* that can break down and utilize SCF (D'Amelio and Sassi 2018). For instance, a study involving adolescent females revealed a positive correlation between SCF intake levels and calcium absorption. Additionally, the proportion of *Parabacteroides* in the GM of adolescent females with high SCF intake was higher compared to the control group (Whisner et al. 2016).

*Bacteroides* genus is a group of Gram-negative bacteria, and LPS on its surface can trigger an inflammatory response when it enters the bloodstream (Geng et al. 2016). The presence of LPS can enhance intestinal permeability (Guo et al. 2013), leading to easier entrance of LPS to bloodstream. Moreover, LPS promotes the differentiation of T cells into Th17 cells (Park et al. 2015), which inhibits osteoblast activity and promotes osteoclast activity (Sato et al. 2006). However, LPS on the surface of *Bacteroides* is about one-thousandth as virulent as the LPS of *Escherichia coli* due to the lack of O-antigen (Weintraub et al. 1985; Zafar and Saier 2021). Nevertheless, certain strains of *Bacteroidetes*, like *Bacteroides fragilis*, can inhibit the human immune response and the differentiation of Th17 cells through Toll-like receptors in the presence of Polysaccharide A (Round et al. 2011). Through pathways such as succinate, *Bacteroides* can produce SCFAs such as acetate and propionate, which induce Tregs responses, thus benefiting bone health (De Martinis et al. 2020; Koh et al. 2016). Yuan and Shen (2021) demonstrated that *Bacteroides vulgatus* can downregulate the expression of serum TNF-α/ Receptor activator of nuclear factor kappa-Β ligand (RANKL) in ovariectomized female mice. This, in turn, leads to a reduction in bone loss and the preservation of bone microarchitecture. Additionally, there is a negative correlation between the number of *Bacteroides* and *Prevotella* and serum leptin levels (Queipo-Ortuno et al. 2013).

***Phylum Actinobacteria***

The VDR promotes bone formation and reduces bone resorption. Studies conducted on vitamin D receptor knockout (Vdr-/-) mice have shown an increased abundance of *Eggerthella* in their GM (Jin et al. 2015). Furthermore, in an epidemiological investigation, researchers discovered a strong association between VDR gene polymorphisms and decreased BMD, as well as an increased risk of osteoporosis. These findings further support the notion that *Eggerthella* may impact host bone metabolism by influencing calcium absorption (Kow et al. 2019).

*Bifidobacterium bifidum* possesses cell surface β-glucan/galactan polysaccharides that can activate Toll-like receptor 2 on intestinal dendritic cells, thereby promoting the production of Tregs (Verma et al. 2018). Additionally, *Bifidobacterium longum BL536* potentially influences the differentiation of Th17 and Tregs by altering serum concentrations of TGF-β (Donkor et al. 2012). By downregulating the expression of NF-κB-linked TNF-α, *Bifidobacterium longum NK49* has shown a protective effect on bone quality in ovariectomized mice (Kim et al. 2019). Apart from its effects on the immune system, *Bifidobacterium longum* also produces SCFAs that can lower the pH in the intestine. This, in turn, promotes calcium absorption and contributes to osteoprotection (Rodrigues et al. 2012).

***Phylum Proteobacteria***

*Proteobacteria* constitutes a prevalent class of bacteria within the gut ecosystem, encompassing several pathogenic species, including *Brucella*, *Neisseria*, *Escherichia*, *Shigella*, and *Salmonella*, characterized by the presence of LPS on their surfaces (Rizzatti et al. 2017). Empirical evidence indicates that an augmented abundance of *Proteobacteria* is often concomitant with ageing and antibiotic-induced perturbations in GM (Biagi et al. 2010; Guss et al. 2017). Clinical investigations have delineated connections between *Proteobacteria* and diverse chronic metabolic diseases (Rizzatti et al. 2017). An animal study (Li et al. 2020b) revealed that the administration of tuna fish powder to osteoporotic mice ameliorated the disease condition by increasing SCFAs production. Accompanied by a significant reduction of *Proteobacteria*. Additionally, exposure to ultraviolet light, while increasing BMD in osteoporotic mice, induced alterations in GM, chiefly a significant diminution in *Proteobacteria* (Cui et al. 2021). Despite animal experiments in diverse disease contexts often showcasing a negative correlation between *Proteobacteria* and BMD, the reviewed studies did not discern substantive differences in *Proteobacteria* proportions between individuals with osteoporosis and those without osteoporosis.

**References**

Akimbekov NS, Digel I, Sherelkhan DK et al(2020) Vitamin D and the host-gut microbiome: a brief overview. Acta Histochem Cytochem 53(3):33-42 https://doi.org/ 10.1267/ahc.20011

Alswat KA (2017) Gender Disparities in Osteoporosis. J Clin Med Res 9(5):382-387. <https://doi.org/10.14740/jocmr2970w>

Aoun A, Darwish F, Hamod N (2020) The Influence of the Gut Microbiome on Obesity in Adults and the Role of Probiotics, Prebiotics, and Synbiotics for Weight Loss. Prev Nutr Food Sci 25(2):113-123. <https://doi.org/10.3746/pnf.2020.25.2.113>

Arpaia N, Campbell C, Fan X et al (2013) Metabolites produced by commensal bacteria promote peripheral regulatory T-cell generation. Nature 504(7480):451-455. <https://doi.org/10.1038/nature12726>

Belzer C, Chia LW, Aalvink S et al (2017) Microbial metabolic networks at the mucus layer lead to diet-independent butyrate and vitamin B12 production by intestinal symbionts. MBio 8(5):e00770-00717. <https://doi.org/10.1128/mBio.00770-17>

Bhardwaj A, Sapra L, Verma B et al (2022) Homocysteine and Bone Health. Homocysteine Metabolism in Health and Disease, Springer, pp 71-95

Biagi E, Nylund L, Candela M et al (2010) Through ageing, and beyond: gut microbiota and inflammatory status in seniors and centenarians. PLoS One 5(5):e10667. <https://doi.org/10.1371/journal.pone.0010667>

Boufker HI, Lagneaux L, Fayyad-Kazan H et al (2011) Role of farnesoid X receptor (FXR) in the process of differentiation of bone marrow stromal cells into osteoblasts. Bone 49(6):1219-1231. https://doi.org/10.1016/j.bone.2011.08.013

Cervantes-Barragan L, Chai JN, Tianero MD et al (2017) Lactobacillus reuteri induces gut intraepithelial CD4(+)CD8alphaalpha(+) T cells. Science 357(6353):806-810. <https://doi.org/10.1126/science.aah5825>

Cheng J, Zhong WL, Zhao JW et al (2022) Alterations in the composition of the gut microbiota affect absorption of cholecalciferol in severe osteoporosis. J Bone Miner Metab 40(3):478-486. <https://doi.org/10.1007/s00774-021-01303-5>

Cho SW, An JH, Park H et al (2013) Positive regulation of osteogenesis by bile acid through FXR. Journal of Bone and Mineral Research 28(10):2109-2121. https://doi.org/ 10.1002/jbmr.1961

Crespel A, De Boisvilliers F, Gros L, Kervran A (1996) Effects of glucagon and glucagon-like peptide-1-(7-36) amide on C cells from rat thyroid and medullary thyroid carcinoma CA-77 cell line. Endocrinology 137(9):3674-3680

Cui J, Fu Y, Yi Z et al (2021) The beneficial effects of ultraviolet light supplementation on bone density are associated with the intestinal flora in rats. Appl Microbiol Biotechnol 105(9):3705-3715. <https://doi.org/10.1007/s00253-021-11282-2>

D'Amelio P, Sassi F (2018) Gut Microbiota, Immune System, and Bone. Calcif Tissue Int 102(4):415-425. <https://doi.org/10.1007/s00223-017-0331-y>

Das M, Cronin O, Keohane DM et al (2019) Gut microbiota alterations associated with reduced bone mineral density in older adults. Rheumatology (Oxford) 58(12):2295-2304. <https://doi.org/10.1093/rheumatology/kez302>

Davie JR (2003) Inhibition of histone deacetylase activity by butyrate. J Nutr 133(7 Suppl):2485S-2493S. <https://doi.org/10.1093/jn/133.7.2485S>

De Martinis M, Ginaldi L, Allegra A et al (2020) The Osteoporosis/Microbiota Linkage: The Role of miRNA. Int J Mol Sci 21(23):8887. <https://doi.org/10.3390/ijms21238887>

Di DS, Li C, Dai Y et al (2021) Integrative Analysis of LGR5/6 Gene Variants, Gut Microbiota Composition and Osteoporosis Risk in Elderly Population. Front Microbiol 12:765008. <https://doi.org/10.3389/fmicb.2021.765008>

Donkor ON, Ravikumar M, Proudfoot O et al (2012) Cytokine profile and induction of T helper type 17 and regulatory T cells by human peripheral mononuclear cells after microbial exposure. Clin Exp Immunol 167(2):282-295. <https://doi.org/10.1111/j.1365-2249.2011.04496.x>

Engen PA, Green SJ, Voigt RM et al (2015) The Gastrointestinal Microbiome: Alcohol Effects on the Composition of Intestinal Microbiota. Alcohol Res 37(2):223-236

Geng S, Chen K, Yuan R et al (2016) The persistence of low-grade inflammatory monocytes contributes to aggravated atherosclerosis. Nat Commun 7(1):13436. <https://doi.org/10.1038/ncomms13436>

Guo S, Al-Sadi R, Said HM et al (2013) Lipopolysaccharide causes an increase in intestinal tight junction permeability in vitro and in vivo by inducing enterocyte membrane expression and localization of TLR-4 and CD14. Am J Pathol 182(2):375-387. <https://doi.org/10.1016/j.ajpath.2012.10.014>

Guss JD, Horsfield MW, Fontenele FF et al (2017) Alterations to the Gut Microbiome Impair Bone Strength and Tissue Material Properties. J Bone Miner Res 32(6):1343-1353. <https://doi.org/10.1002/jbmr.3114>

He J, Xu S, Zhang B et al (2020) Gut microbiota and metabolite alterations associated with reduced bone mineral density or bone metabolic indexes in postmenopausal osteoporosis. Aging 12(9):8583-8604

Jin D, Wu S, Zhang YG et al (2015) Lack of Vitamin D Receptor Causes Dysbiosis and Changes the Functions of the Murine Intestinal Microbiome. Clin Ther 37(5):996-1009 e1007. <https://doi.org/10.1016/j.clinthera.2015.04.004>

Jones ML, Martoni CJ, Prakash S (2013) Oral supplementation with probiotic L. reuteri NCIMB 30242 increases mean circulating 25-hydroxyvitamin D: a post hoc analysis of a randomized controlled trial. J Clin Endocrinol Metab 98(7):2944-2951. <https://doi.org/10.1210/jc.2012-4262>

Keirns BH, Lucas EA, Smith BJ (2020) Phytochemicals affect T helper 17 and T regulatory cells and gut integrity: implications on the gut-bone axis. Nutr Res 83:30-48. <https://doi.org/10.1016/j.nutres.2020.08.006>

Kim D-E, Kim J-K, Han S-K et al (2019) Lactobacillus plantarum NK3 and Bifidobacterium longum NK49 alleviate bacterial vaginosis and osteoporosis in mice by suppressing NF-κ B-Linked TNF-α expression. Journal of medicinal food 22(10):1022-1031. <https://doi.org/10.1089/jmf.2019.4419>

Koh A, De Vadder F, Kovatcheva-Datchary P et al (2016) From dietary fiber to host physiology: short-chain fatty acids as key bacterial metabolites. Cell 165(6):1332-1345. <https://doi.org/10.1016/j.cell.2016.05.041>

Kong Y-Y, Feige U, Sarosi I et al (1999) Activated T cells regulate bone loss and joint destruction in adjuvant arthritis through osteoprotegerin ligand. Nature 402(6759):304-309. <https://doi.org/10.1038/46303>

Kow M, Akam E, Singh P et al (2019) Vitamin D receptor (VDR) gene polymorphism and osteoporosis risk in White British men. Annals of Human Biology 46(5):430-433. https://doi.org/10.1080/03014460.2019.1659851

Laird E, Ward M, McSorley E et al (2010) Vitamin D and bone health; Potential mechanisms. Nutrients 2(7):693-724. https://doi.org/ 10.3390/nu2070693

Lange K, Buerger M, Stallmach A et al (2016) Effects of Antibiotics on Gut Microbiota. Dig Dis 34(3):260-268. <https://doi.org/10.1159/000443360>

Lei M, Hua LM, Wang DW (2016) The effect of probiotic treatment on elderly patients with distal radius fracture: a prospective double-blind, placebo-controlled randomised clinical trial. Benef Microbes 7(5):631-637. <https://doi.org/10.3920/BM2016.0067>

Ley RE, Peterson DA, Gordon JI (2006) Ecological and evolutionary forces shaping microbial diversity in the human intestine. Cell 124(4):837-848. <https://doi.org/10.1016/j.cell.2006.02.017>

Li H, Wallin M, Barregard L et al (2020a) Smoking‐induced risk of osteoporosis is partly mediated by cadmium from tobacco smoke: The MrOS Sweden Study. J Bone Miner Res 35(8):1424-1429. <https://doi.org/10.1002/jbmr.4014>

Li J, Yang M, Lu C et al (2020b) Tuna Bone Powder Alleviates Glucocorticoid-Induced Osteoporosis via Coregulation of the NF-kappaB and Wnt/beta-Catenin Signaling Pathways and Modulation of Gut Microbiota Composition and Metabolism. Mol Nutr Food Res 64(5):e1900861. <https://doi.org/10.1002/mnfr.201900861>

Li J-Y, Chassaing B, Tyagi AM et al (2016) Sex steroid deficiency–associated bone loss is microbiota dependent and prevented by probiotics. The Journal of clinical investigation 126(6):2049-2063. <https://doi.org/10.1172/JCI86062>

Li J-Y, Yu M, Pal S et al (2020c) Parathyroid hormone–dependent bone formation requires butyrate production by intestinal microbiota. The Journal of clinical investigation 130(4):1767-1781. https://doi.org/ 10.1172/JCI133473

Liu Z, Habener JF (2008) Glucagon-like peptide-1 activation of TCF7L2-dependent Wnt signaling enhances pancreatic beta cell proliferation. Journal of Biological Chemistry 283(13):8723-8735. https://doi.org/10.1074/jbc.M706105200

Lopetuso LR, Scaldaferri F, Petito V et al (2013) Commensal Clostridia: leading players in the maintenance of gut homeostasis. Gut Pathog 5(1):23. <https://doi.org/10.1186/1757-4749-5-23>

Lucas S, Omata Y, Hofmann J et al (2018) Short-chain fatty acids regulate systemic bone mass and protect from pathological bone loss. Nat Commun 9(1):55. <https://doi.org/10.1038/s41467-017-02490-4>

Luo C, Wang L, Sun C et al (2011) Estrogen enhances the functions of CD4+ CD25+ Foxp3+ regulatory T cells that suppress osteoclast differentiation and bone resorption in vitro. Cellular & molecular immunology 8(1):50-58. https://doi.org/10.1038/cmi.2010.54

Lv J, Zhao H, Yu Y et al (2021) Profile and gene functional analysis of gut microbiota in women with postmenopausal osteoporosis. 41 (Chinese Journal of Microbiology and Immunology). <https://doi.org/10.3760/cma.j.cn112309-20210425-00134>

Makishima M, Lu TT, Xie W et al (2002) Vitamin D receptor as an intestinal bile acid sensor. Science 296(5571):1313-1316. https://doi.org/ 10.1126/science.1070477

Martínez I, Lattimer JM, Hubach KL et al (2013) Gut microbiome composition is linked to whole grain-induced immunological improvements. The ISME journal 7(2):269-280. https://doi.org/ 10.1038/ismej.2012.104

McCabe L, Britton RA, Parameswaran N (2015) Prebiotic and Probiotic Regulation of Bone Health: Role of the Intestine and its Microbiome. Curr Osteoporos Rep 13(6):363-371. <https://doi.org/10.1007/s11914-015-0292-x>

Narva M, Nevala R, Poussa T et al (2004) The effect of Lactobacillus helveticus fermented milk on acute changes in calcium metabolism in postmenopausal women. Eur J Nutr 43(2):61-68. <https://doi.org/10.1007/s00394-004-0441-y>

Nilsson AG, Sundh D, Backhed F et al (2018) Lactobacillus reuteri reduces bone loss in older women with low bone mineral density: a randomized, placebo-controlled, double-blind, clinical trial. J Intern Med 284(3):307-317. <https://doi.org/10.1111/joim.12805>

Nishida A, Inoue R, Inatomi O et al (2018) Gut microbiota in the pathogenesis of inflammatory bowel disease. Clin J Gastroenterol 11(1):1-10. <https://doi.org/10.1007/s12328-017-0813-5>

Page MJ, McKenzie JE, Bossuyt PM et al (2021) The PRISMA 2020 statement: an updated guideline for reporting systematic reviews. Syst Rev 10(1):1-11. <https://doi.org/10.1136/bmj.n71>

Park JH, Jeong SY, Choi AJ et al (2015) Lipopolysaccharide directly stimulates Th17 differentiation in vitro modulating phosphorylation of RelB and NF-kappaB1. Immunol Lett 165(1):10-19. <https://doi.org/10.1016/j.imlet.2015.03.003>

Plassais J, Gbikpi-Benissan G, Figarol M et al (2021) Gut microbiome alpha-diversity is not a marker of Parkinson’s disease and multiple sclerosis. Brain commun 3(2):fcab113. <https://doi.org/10.1093/braincomms/fcab113>.

Pushalkar S, Li X, Kurago Z et al (2014) Oral microbiota and host innate immune response in bisphosphonate-related osteonecrosis of the jaw. Int J Oral Sci 6(4):219-226. <https://doi.org/10.1038/ijos.2014.46>

Qin Q, Yan S, Yang Y et al (2021) The Relationship Between Osteoporosis and Intestinal Microbes in the Henan Province of China. Front Cell Dev Biol 9. <https://doi.org/10.3389/fcell.2021.752990>.

Queipo-Ortuno MI, Seoane LM, Murri M et al (2013) Gut microbiota composition in male rat models under different nutritional status and physical activity and its association with serum leptin and ghrelin levels. PLoS One 8(5):e65465. <https://doi.org/10.1371/journal.pone.0065465>

Reese AT, Dunn RR (2018) Drivers of Microbiome Biodiversity: A Review of General Rules, Feces, and Ignorance. mBio 9(4):e01294-01218. <https://doi.org/10.1128/mBio.01294-18>

Reigstad CS, Salmonson CE, Rainey III JF et al (2015) Gut microbes promote colonic serotonin production through an effect of short-chain fatty acids on enterochromaffin cells. The FASEB Journal 29(4):1395. <https://doi.org/10.1096/fj.14-259598>.

Rettedal EA, Ilesanmi‐Oyelere BL, Roy NC et al (2021) The gut microbiome is altered in postmenopausal women with osteoporosis and osteopenia. JBMR plus 5(3):e10452. <https://doi.org/10.1002/jbm4.10452>.

Ríos-Covián D, Ruas-Madiedo P, Margolles A et al (2016) Intestinal short chain fatty acids and their link with diet and human health. Front Microbiol 7:185. https://doi.org/ 10.3389/fmicb.2016.00185

Rizzatti G, Lopetuso LR, Gibiino G et al (2017) Proteobacteria: A Common Factor in Human Diseases. Biomed Res Int 2017:9351507. <https://doi.org/10.1155/2017/9351507>

Rodrigues FC, Castro AS, Rodrigues VC et al (2012) Yacon flour and Bifidobacterium longum modulate bone health in rats. J Med Food 15(7):664-670. <https://doi.org/10.1089/jmf.2011.0296>

Round JL, Lee SM, Li J, Tran G et al (2011) The Toll-like receptor 2 pathway establishes colonization by a commensal of the human microbiota. Science 332(6032):974-977. <https://doi.org/10.1126/science.1206095>

Sapra L, Dar HY, Bhardwaj A et al (2021) Lactobacillus rhamnosus attenuates bone loss and maintains bone health by skewing Treg-Th17 cell balance in Ovx mice. Sci Rep 11(1):1-18. https://doi.org/10.1038/s41598-020-80536-2

Sato K, Suematsu A, Okamoto K et al (2006) Th17 functions as an osteoclastogenic helper T cell subset that links T cell activation and bone destruction. J Exp Med 203(12):2673-2682. <https://doi.org/10.1084/jem.20061775>.

Smith PM, Howitt MR, Panikov N et al (2013) The microbial metabolites, short-chain fatty acids, regulate colonic Treg cell homeostasis. Science 341(6145):569-573. <https://doi.org/10.1126/science.1241165>

Taylor A, Verhagen J, Blaser K et al (2006) Mechanisms of immune suppression by interleukin‐10 and transforming growth factor‐β: the role of T regulatory cells. Immunology 117(4):433-442. https://doi.org/10.1111/j.1365-2567.2006.02321.x

Tyagi AM, Yu M, Darby TM et al (2018) The Microbial Metabolite Butyrate Stimulates Bone Formation via T Regulatory Cell-Mediated Regulation of WNT10B Expression. Immunity 49(6):1116-1131 e1117. <https://doi.org/10.1016/j.immuni.2018.10.013>

Verma R, Lee C, Jeun EJ et al (2018) Cell surface polysaccharides of Bifidobacterium bifidum induce the generation of Foxp3(+) regulatory T cells. Sci Immunol 3(28):eaat6975. <https://doi.org/10.1126/sciimmunol.aat6975>

Wang B, Zhao H, Gao W et al (2017a) Diversity analysis of intestinal microbial flora in osteoporosis patients. Chinese Journal of Osteoporosis/Zhongguo Guzhi Shusong Zazhi 23(6). <https://doi.org/10.3969/j.issn.1006.7108.2017.06.004>

Wang J, Thingholm LB, Skiecevičienė J et al (2016) Genome-wide association analysis identifies variation in vitamin D receptor and other host factors influencing the gut microbiota. Nat Genet 48(11):1396-1406. https://doi.org/10.1038/ng.3695

Wang J, Wang Y, Gao W et al (2017b) Diversity analysis of gut microbiota in osteoporosis and osteopenia patients. PeerJ 5:e3450. <https://doi.org/10.7717/peerj.3450>

Wei M, Li C, Dai Y et al (2021) High-Throughput Absolute Quantification Sequencing Revealed Osteoporosis-Related Gut Microbiota Alterations in Han Chinese Elderly. Front Cell Infect Microbiol 11:630372. <https://doi.org/10.3389/fcimb.2021.630372>

Weintraub A, Larsson BE, Lindberg AA (1985) Chemical and immunochemical analyses of Bacteroides fragilis lipopolysaccharides. Infect Immun 49(1):197-201. <https://doi.org/10.1128/iai.49.1.197-201.1985>

Whisner CM, Martin BR, Nakatsu CH et al (2016) Soluble Corn Fiber Increases Calcium Absorption Associated with Shifts in the Gut Microbiome: A Randomized Dose-Response Trial in Free-Living Pubertal Females. J Nutr 146(7):1298-1306. <https://doi.org/10.3945/jn.115.227256>

Worsley SF, Davies CS, Mannarelli M-E et al (2021) Gut microbiome composition, not alpha diversity, is associated with survival in a natural vertebrate population. Anim Microbiome 3(1):1-18. <https://doi.org/10.1186/s42523-021-00149-6>.

Xu Z, Xie Z, Sun J et al (2020) Gut Microbiome Reveals Specific Dysbiosis in Primary Osteoporosis. Front Cell Infect Microbiol 10:160. <https://doi.org/10.3389/fcimb.2020.00160>

Yadav VK, Oury F, Suda N et al (2009) A serotonin-dependent mechanism explains the leptin regulation of bone mass, appetite, and energy expenditure. Cell 138(5):976-989. <https://doi.org/10.1016/j.cell.2009.06.051>

Yan J, Herzog JW, Tsang K et al (2016) Gut microbiota induce IGF-1 and promote bone formation and growth. Proc Natl Acad Sci U S A 113(47):E7554-E7563. <https://doi.org/10.1073/pnas.1607235113>

Yatsunenko T, Rey FE, Manary MJ et al (2012) Human gut microbiome viewed across age and geography. Nature 486(7402):222-227. <https://doi.org/10.1038/nature11053>

Yuan S, Shen J (2021) Bacteroides vulgatus diminishes colonic microbiota dysbiosis ameliorating lumbar bone loss in ovariectomized mice. Bone 142:115710. <https://doi.org/10.1016/j.bone.2020.115710>

Yuan Y, Yang J, Zhuge A et al (2022) Gut microbiota modulates osteoclast glutathione synthesis and mitochondrial biogenesis in mice subjected to ovariectomy. Cell Prolif 55(3):e13194. <https://doi.org/10.1111/cpr.13194>

Zafar H, Saier MH, Jr. (2021) Gut Bacteroides species in health and disease. Gut Microbes 13(1):1-20. <https://doi.org/10.1080/19490976.2020.1848158>

**Identification of studies via databases and registers**

Records removed before screening:

Duplicate English records removed (n = 419)

Duplicate Chinese records removed (n = 122)

Records identified from:

English databases (n = 4)

Registers (n = 1409)

Chinese databases (n = 4)

Registers (n = 332)

**Identification**

Records excluded (n = 974+205) with reasons:

In vitro/ Animal studies/ Case reports/ Meeting abstracts/ Reviews/ Book or chapters/ Editorials/ Not related

Records screened from:

English databases (n = 990)

Chinese databases (n = 210)

Reports sought for retrieval from:

English databases (n = 20)

Chinese databases (n = 5)

(n =16 )

Reports not retrieved

(n = 0)

**Screening**

Reports assessed for eligibility

(n = 25)

Reports excluded:

Reason 1 (n = 2)

Reason 2 (n = 3)

Reason 3 (n = 5)

Reason 4 (n = 2)

Reason 5 (n = 2)

Studies included in review

(n = 10)

**Included**

Reason 1: Articles using the same data.

Reason 2: Not classified by osteoporosis patients, healthy controls.

Reason 3: Irrelevant study.

Reason 4: Study subjects are not adults or neither elderly patients nor post-menopausal patients with osteoporosis.

Reason 5: Using data from public database.

Fig. S1 PRISMA Flow Diagram

**Table S1 General Character of Reviewed Literature**

| **Literature** | **Study design** | **Location** | **Number and age of participants** | **Inclusion criteria** | **Exclusion criteria** |
| --- | --- | --- | --- | --- | --- |
| **(Wang et al. 2017a)** | Cross sectional | China, Xi’an | OP: 7 (68. 20±5.02, all female)  HC: 7 (66.80±4.60, all female) | Patients admitted to Xi’an Red Cross Hospital from May to August 2016. Subjects were free of comorbid gastrointestinal, systemic diseases such as hypertension and diabetes mellitus. Subjects were required to be free of antibiotics, steroids, herbal preparations, micro-ecological preparations, probiotics such as yoghurt, etc. for one week prior to specimen collection | N/A |
| **(Lv et al. 2021)** | Cross sectional | China, Xi’an | OP: 24 (61.0±6.6, all female)  HC: 9 (55.3±3.6, all female) | Postmenopausal women attending Xi'an Red Cross Hospital from November 2018 to October 2019. | People with metabolic bone disease, neuroendocrine or immune disorders, chronic systemic diseases, etc.; taking anti-osteoporosis or drugs that affect bone metabolism; being bedridden for more than 3 months recently, or excessive smoking and alcohol consumption; taking any antibiotics, micro-ecological regulators and herbal preparations within one month prior to the collection of the stool specimen. |
| **(Rettedal et al. 2021)** | Cross sectional | New Zealand | OP: 18 (64.6±5.66, all female)  HC: 26 (62.5±4.54, all female) | 68 postmenopausal women aged 54 to 81 years participated in phase II of the “Bugs‘n’Bones” study that took place in the Human Nutrition Unit of Massey University, Palmerston North between October 17, 2017 and March 6, 2018. The inclusion criteria required menopause of at least 5 years based on no menstruation. | Exclusion criteria included the presence of any systemic disease, food intolerances that affect the gut, smokers, high intake of alcohol (>2 units/day), or use of antibiotics within 3 months of the study. Participants with significant weight loss or weight gain within the past year were also excluded. No participants were undergoing medical treatment for osteoporosis or osteopenia. |
| **(He et al. 2020)** | Cross sectional | China, Xiamen | OP: 42 (59.69±5.51, all female)  HC: 31 (57.35±3.98, all female) | No menstruation for at least 12 months after the last menopause was considered a postmenopausal status. | Participants with cancer, kidney disease, metabolic or genetic bone disease, digestive system disease (inflammatory bowel disease, hepatic disease, constipation, previous partial or total colectomy),  psychiatric disease (e.g., schizophrenia, depression,  or cognitive impairment), or use of antibiotics in the past 3 months or patients using medications (e.g., estrogen, glucocorticoids, diphosphonate, teriparatide or denosumab) that might influence bone metabolism were excluded. |
| **(Di et al. 2021)** | Cross sectional | China, Wuhan | OP: 77 (67.0±6.7, 56 female and 21 male)  HC: 103(66.1±7.2, 56 female and 47 male) | 1,168 Han Chinese participants aged ≥60 years recruited at two communities in Wuhan city and the Wuhan Union Hospital during 2016–2018. | The exclusion criteria were as follows: (1) with other endocrine diseases (e.g., hyperthyroidism, hypothyroidism, etc.) that influences bone metabolism; (2) of surgical menopause (i.e., hysterectomy and/or ovariectomy); and (3) taking medicines affecting bone health such as hormones. Moreover, individuals were further excluded before stool collection if they met the following criterion: (1) use of antibiotics within 1 month before fecal sample collection; and (2) with prevalent diseases of diabetes and gastrointestinal diseases. |
| **(Wei et al. 2021)** | Cross sectional | China, Wuhan | OP: 44 (69.73±5.47, 35 female and 9 male)  HC: 64 (67.84±4.83, 32 female and 32 male) | All participants were recruited at Union Hospital of Tongji Medical College in Wuhan City from 2018 to 2019. Adults older than 60 years or postmenopausal women with  natural menopause were included in this study. | Individuals taking antibiotics or hormones within the past month before stool collection were excluded. Participants with disease history of hyperthyroidism or hypothyroidism, and prevalent gastrointestinal, renal, or osteoarthritis diseases were also excluded. Women with hysterectomy and ovariectomies were excluded. |
| **(Wang et al. 2017b)** | Cross sectional | China, Xi’an | OP: 6 (70.00±7.77, 5 female and 1 male)  HC: 6 (64.80±5.93, 5 female and 1 male) | Participants in this study were recruited from Hong Hui Hospital, Xi’an Jiaotong University,  Xi’an, China. | We further excluded all patients with any malignancy, chronic liver disease, heart disease, kidney disease, or diabetes. |
| **(Das et al. 2019)** | Cohort | Ireland | OP: 61 (64.84±5.28, 54 female and 7 male)  HC: 60 (63.57±5.73, 47 female and 13 male) | Adult female and male subjects, aged 55-75 years, were recruited from the bone densitometry  unit at Cork University Hospital, Cork, Ireland. | Individuals with a known history of alcohol abuse, participation in an investigational drug trial in the 30 days before enrolment, use of antibiotics in the 3 months prior to bone density measurement, and previous partial or total colectomy were excluded. |
| **(Qin et al. 2021)** | Cohort | China, Zhengzhou | OP: 11 (58.72 ± 4.06, 5 female and 6 male)  HC: 18 (62.00 ± 7.33, 4 female and 14 male) * | Study participants aged 55–75 years were recruited at First Affiliated Hospital of Zhengzhou University hospital from January 2018 to July 2019. The inclusion criterion was health checkups over 55–75 years of age. | 1. patients with metal implants within the upper abdomen scan; 2) alcohol abuse or smokers, history of surgery within 1-year, chronic diseases such as hypertension, diabetes, coronary heart disease, or tumors; 3) diseases and drug treatments that affect BMD and antibiotic use; and 4) participants who have had previous fracture history (accidental fracture and fragility fractures). |
| **(Xu et al. 2020)** | Cross sectional | China, Zhengzhou | OP: 48 (57.50±10.15, 30 female and 18 male)  HC: 48 (60.23±11.43, 29 female and 19 male) | Orthopedic inpatients in First Affiliated Hospital of Zhengzhou University hospital in 2019  were prospectively collected in this study. | (1) secondary osteoporosis; (2) incomplete case data or patients and their families could not cooperate to complete the questionnaire survey; (3) age < 40 years old; (4) antibiotics, probiotics, probiotics, or symbiosis in the 2 months before stool samples were collected; (5) patients with severe malnutrition, infection, drug use, or alcohol abuse; (6) patients with digestive system diseases; (7) patients with severe systemic diseases, tumors, or other serious primary diseases; (8) previous lumbar and hip surgery; (9) fecal samples were not up to standard. |

* Qin (2021) included 59 HC and 30 OP in all which were further divided into training dataset and validation dataset, but only the statistic for the training data set can be retrieved. N/A: Not mentioned.

**Table S2 Confounding Variables Control across Literature on Postmenopausal Osteoporosis**

| **Literature** | **Confounding variables** | | | | | | | |
| --- | --- | --- | --- | --- | --- | --- | --- | --- |
|  | **Age** | **BMI** | **Bowel diseases** | **Antibiotic** | **Bone metabolic medicine** | **Prebiotics and probiotic** | **Alcohol** | **Smoking** |
| **(Wang et al. 2017a)** | N | N | Y | Y | N | Y | N | N |
| **(Lv et al. 2021)** | N | N | Y | Y | Y | Y | Y | Y |
| **(Rettedal et al. 2021)** | Y | N | Y | Y | Y | Y | Y | Y |
| **(He et al. 2020)** | Y | Y | Y | Y | Y | Y | N | N |

Y: Controlled. N: Uncontrolled.

**Table S2 Confounding Variables Control across Literature on Senile Osteoporosis**

| **Literature** | **Confounding variables** | | | | | | | | | | | | |
| --- | --- | --- | --- | --- | --- | --- | --- | --- | --- | --- | --- | --- | --- |
|  | **Age** | **BMI** | | **Gender** | | **Related disease** | **Antibiotic** | **Bone metabolic medicine** | **Prebiotics and probiotic** | **Alcohol** | | **Smoking** | |
| **(Di et al. 2021)*** | Y | | N | | N | Y | Y | Y | N | | Y | | N |
| **(Wei et al. 2021)*** | Y | | N | | N | Y | Y | N | N | | Y | | Y |
| **(Wang et al. 2017b)** | Y | | N | | Y | Y | Y | Y | Y | | N | | N |
| **(Das et al. 2019)**** | Y | | N | | Y | N | Y | N | N | | N | | N |
| **(Qin et al. 2021)** | Y | | Y | | Y | Y | Y | Y | N | | Y | | Y |
| **(Xu et al. 2020)** | Y | | Y | | Y | Y | Y | N | Y | | Y | | N |

Y: Controlled. N: Uncontrolled.

* Ridge regression was used to adjust for age, gender, smoking and BMI. ** Univariate general linear mixed model regression with a negative binomial distribution was used to adjust for BMI, medications and vitamin D levels.

**Table S3 Methodological Character of Literature**

| **Literature** | **Fecal microbiome** | | | | | | | | **Bone mass density** |
| --- | --- | --- | --- | --- | --- | --- | --- | --- | --- |
|  | **Premier** | **Variable region** | **Truncated length** | **Sequencing platform** | **Pipeline** | **Clustering approach** | **Reference database** | **Abundance quantification** |  |
| **(Wang et al. 2017a)** | 338F/806R | V3-V4 | N/A | Illumina MiSeq | N/A | OTUs | RDP | Relative | N/A |
| **(Lv et al. 2021)** | 338F/806R | V3-V4 | 250bp | Illumina NovaSeq 6000 | QIIME (v1.8.0) | OTUs | NCBI/KEGG | Relative | DXA |
| **(He et al. 2020)** | 341F/806R | V3-V4 | 250bp | Illumina HiSeq 2500 | Unoise3 of usearch | OTUs | Silva | Relative | DXA |
| **(Di et al. 2021)** | 341F/805R | V3-V4 | 250bp | Illumina MiSeq | Mothur | OTUs | RDP | Relative | DXA |
| **(Wei et al. 2021)** | 341F/805R | V3-V4 | 250bp | Illumina NovaSeq 6000 | Mothur (v1.41.1) | OTUs | RDP (v11.5) | Absolute and Relative | DXA |
| **(Wang et al. 2017b)** | 338F/806R | V3-V4 | 250bp | Illumina MiSeq | QIIME (v1.7) | OTUs | Silva (SSU123) | Relative | DXA |
| **(Das et al. 2019)** | 341F/805R | V3-V4 | 250bp | Illumina MiSeq | QIIME (v1.9.1) | OTUs | RDP (v11.4) | Relative | DXA |
| **Xu (2020)** | 515F/806R | V3-V4 | N/A | Ion S5™ XL System | Mothur | OTUs | SILVA132 SSUrRNA | Relative | DXA |
| **(Rettedal et al. 2021)** | Shotgun metagenomic sequencing | | | | | | | Relative | DXA |
| **(Qin et al. 2021)** | Shotgun metagenomic sequencing | | | | | | | Relative | QCT |

N/A: Unmentioned in the original article

**Table S4 Quality Rating**

|  | **Q1** | **Q2** | **Q3** | **Q4** | **Q5** | **Q6** | **Q7** | **Q8** | **Q9** | **Q10** | **Q11** | **Q12** | **Q13** | **Q14** | **Quality rating** | **Notes** |
| --- | --- | --- | --- | --- | --- | --- | --- | --- | --- | --- | --- | --- | --- | --- | --- | --- |
| **(Wang et al. 2017a)** | Y | Y | N/A | Y | N | N | N | N/A | CD | N/A | Y | N/A | N/A | N | Poor | Didn’t report pipeline choice and setting; Confounding factors like age, BMI, bowel diseases, bone metabolic medicine, alcohol and cigarette consumption aren't controlled. Also sample size is limited. |
| **(Lv et al. 2021)** | Y | Y | N/A | Y | N | N | N | N/A | N | N/A | Y | N/A | N/A | N | Poor | Using NCBI database as reference database which may cause bias. Confounding variants (age and BMI) aren’t controlled with limited sample size. |
| **(Rettedal et al. 2021)** | Y | Y | N/A | Y | N | N | N | N/A | Y | N/A | Y | N/A | N/A | N | Fair | BMI isn’t controlled with a relatively small sample size make the results less convincing. |
| **(He et al. 2020)** | Y | Y | N/A | Y | N | N | N | N/A | Y | N/A | Y | N/A | N/A | N | Fair | Alcohol and cigarette consumption aren't controlled |
| **(Di et al. 2021)** | Y | Y | N/A | Y | N | N | N | N/A | Y | N/A | Y | N/A | N/A | N | Fair | Prebiotics and probiotics usage are not controlled. |
| **(Wei et al. 2021)** | Y | Y | N/A | Y | N | N | N | N/A | Y | N/A | Y | N/A | N/A | Y | Good |  |
| **(Wang et al. 2017b)** | Y | Y | N/A | Y | N | N | N | N/A | Y | N/A | Y | N/A | N/A | N | Poor | BMI, alcohol and cigarette consumption aren't controlled. Limited sample size. |
| **(Das et al. 2019)** | Y | Y | N/A | Y | N | N | N | N/A | Y | N/A | Y | N/A | N/A | Y | Good |  |
| **(Qin et al. 2021)** | Y | Y | N/A | Y | N | N | N | N/A | Y | N/A | Y | N/A | N/A | N | Fair | Prebiotics and probiotics usage are not controlled. |
| **(Xu et al. 2020)** | Y | Y | N/A | Y | N | N | N | N/A | Y | N/A | Y | N/A | N/A | N | Fair | Bone metabolic medicine usage and smoking habit aren’t controlled. |

CD: Cannot determine; N/A: Not applicable; Y: Yes; N: No.

**Table S5 Alpha Diversity Summery**

|  | **Shannon** | | **Simpson** | | **Chao1** | | **Ace** | |
| --- | --- | --- | --- | --- | --- | --- | --- | --- |
|  | **OP** | **HC** | **OP** | **HC** | **OP** | **HC** | **OP** | **HC** |
| **(Wang et al. 2017a)** | H* | L | L | H* | H | L | H | L |
| **(Rettedal et al. 2021)** | H* | L | H* | L | N/A | N/A | N/A | N/A |
| **(He et al. 2020)** | L | H | N/A | N/A | N/A | N/A | N/A | N/A |
| **(Di et al. 2021)** | L | H* | H* | L | L | H | L | H |
| **(Wei et al. 2021)** | L | H* | L | H* | H* | L | N/A | N/A |
| **(Wang et al. 2017b)** | H | L | H | L | H | L | H | L |
| **(Das et al. 2019)** | H* | L | N/A | N/A | H* | L | N/A | N/A |
| **(Xu et al. 2020)** | N/A | N/A | N/A | N/A | H | L | H | L |

H: High; L: Low; N/A: Not mentioned *P>0.05

**Table S6 Beta Diversity**

| **Literature** | **Reported beta diversity** |
| --- | --- |
| **(Wang et al. 2017a)** | UniFrac analysis indicated that the intestinal flora of PO patients was different from that of HC group. |
| **(Lv et al. 2021)** | N/A |
| **(Rettedal et al. 2021)** | The PCoA plot did not show an obvious separation between groups. PERMANOVA showed HC versus OP (p = 0.010) indicated significant differences. To look at the potential influence of intragroup variation on the PERMANOVA analysis, PERMDISP was performed. The results were not statistically significant, indicating the PERMANOVA differences were not caused by greater intragroup variability. |
| **(He et al. 2020)** | PCA, PCoA and PERMANOVA based on OTU abundances and found significant differences among the three groups. (Only marginally significant differences between the PMO and control conditions were found.) |
| **(Di et al. 2021)** | The PCoA of weighted UniFrac distance showed a significant difference between the OP and control groups. The PC1 explained 46.64% ofvariation and the PC2 explained 10.53%. The PERMANOVA analysis also revealed that the two groups had a significant difference in beta diversity (F = 3.413, P = 1.0 × 10^−4^). |
| **(Wei et al. 2021)** | PCoA results based on weighted Unifrac matrix clustering and PERMANOVA test results revealed achieving-significance between-group differences of microbial beta diversity (R2 = 0.033, P value = 0.022). |
| **(Wang et al. 2017b)** | Unweighted UniFrac analysis indicated that hierarchical clustering and principal coordinate analysis (PCoA) could discriminate the NC samples from OP samples. |
| **(Das et al. 2019)** | Based on principal coordinate analysis on different beta-diversity measures, Axes 1 and 2 explained 11-17% and 8-13% of variance, respectively. |
| **(Qin et al. 2021)** | To compare the species-level beta diversity of OP and control groups, the Hellinger, Jensen–Shannon divergence (JSD), Bray, and Spearmen distances were calculated. This analysis revealed no significant difference in the species-level beta diversity between OP and control groups. |
| **(Xu et al. 2020)** | The diversity of Beta was analyzed based on Weighted Unifrac and Unweighted Unifrac. Anosim analysis (R = 0.15, P < 0.001), permutational MANOVA (R = 0.06, P < 0.001) suggested that there were significant differences between the two groups. Importantly, there was also a illustrious discrepancy in the composition of gut microbiome between the OP group and the HC group (MRPP, A = 0.0314, observed-delta = 0.7193, expected-delta = 0.7426, P = 0.001) in theBray-Curtis-based PCA diagram. |

**Table S7 Taxonomic Diversity**

|  | Bacteroidetes | | Gemmatimonadetes | | Chloroflexi | | Firmicutes | | Erysipelotrichia | | Ruminococcaceae | | Porphyromonadaceae | | Bacteroidaceae | | Lachnospiraceae | | Eggerthella | | Faecalibacterium | | Parabacteroides | | Dialister | | Veillonella | | Subdoligranulum | | Blautia | | Lactobacillus | | Bacteroides | | Ruminococcaceae UCG002 | |
| --- | --- | --- | --- | --- | --- | --- | --- | --- | --- | --- | --- | --- | --- | --- | --- | --- | --- | --- | --- | --- | --- | --- | --- | --- | --- | --- | --- | --- | --- | --- | --- | --- | --- | --- | --- | --- | --- | --- |
|  | OP | HC | OP | HC | OP | HC | OP | HC | OP | HC | OP | HC | OP | HC | OP | HC | OP | HC | OP | HC | OP | HC | OP | HC | OP | HC | OP | HC | OP | HC | OP | HC | OP | HC | OP | HC | OP | HC |
| (Wang et al. 2017a) | / | / | H | L | H | L | / | / | / | / | / | / | / | / | / | / | / | / | / | / | / | / | / | / | / | / | / | / | / | / | / | / | / | / | / | / | / | / |
| (Lv et al. 2021) | / | / | / | / | / | / | / | / | H | L | / | / | / | / | / | / | L | H | / | / | / | / | / | / | / | / | / | / | / | / | / | / | / | / | / | / | / | / |
| (Rettedal et al. 2021) | / | / | / | / | / | / | / | / | / | / | / | / | / | / | H | L | / | / | / | / | / | / | / | / | / | / | / | / | / | / | / | / | / | / | H | L | / | / |
| (He et al. 2020) | L | H | / | / | / | / | / | / | / | / | / | / | / | / | / | / | / | / | / | / | / | / | H | L | / | / | / | / | / | / | L | H | H | L | / | / | / | / |
| (Di et al. 2021) | H1 | L | / | / | / | / | L | H2 | / | / | ND3 | ND | ND4 | ND | H5 | L | L | H6 | / | / | / | / | ND8 | ND | / | / | / | / | / | / | / | / | ND8 | ND | H11 | L | / | / |
| (Wei et al. 2021) | H | L | / | / | / | / | / | / | / | / | / | / | / | / | / | / | ND4/7 | ND | H7 | L | / | / | H | L | / | / | ND9 | ND | / | / | / | / | H7 | L | H | L | / | / |
| (Wang et al. 2017b) | L | H | H | L | H | L | H | L | / | / | / | / | / | / | / | / | / | / | / | / | ND | ND | H | L | ND | ND | / | / | ND | ND | H | L | / | / | ND | ND | H | L |
| (Das et al. 2019) | ND | ND | / | / | / | / | ND | ND | / | / | / | / | / | / | / | / | / | / | H | L | ND | ND | / | / | ND | ND | L | H | / | / | ND | ND | ND10 | ND | ND | ND | ND | ND |
| (Qin et al. 2021) | / | / | / | / | / | / | / | / | / | / | / | / | L | H | / | / | / | / | / | / | / | / | L | H | / | / | / | / | / | / | / | / | / | / | / | / | / | / |
| (Xu et al. 2020) | H | L | / | / | / | / | ND | ND | L | H | H | L | / | / | / | / | L | H | / | / | H | L | / | / | H | L | / | / | L | H | L | H | / | / | ND | ND | / | / |

Notes: OP: Osteoporosis group; HC: Health control group; H: High; L: Low; ND: No significant difference was found between groups; /: Not mentioned. 1: p_Bacteroidetes is negatively correlated with LS T-score LS Z-score;

2: p_Firmicutes is positively correlated with Hip T-score;

3: f_Ruminococcaceae correlated positively with the BMDs and T-scores among all subjects but after regression no difference;

4: f_Porphyromonadaceae, f_Lachnospiraceae enriched in OP before regression;

5: f_Bacteroidaceae is negatively correlated with LS T-score, LS Z-score, FN BMD, FN Z-score and Hip Z-score;

6: f_Lachnospiraceae is enriched in HC but is negatively correlated with Hip T-score;

7: Found in absolute abundance not relative abundance;

8: Enriched in OP and the difference between groups is significant before regression;

9: g_Veillonella is enriched in HC and the difference between groups is significant before regression. The result is found in absolute abundance not relative abundance;

10: g_Lactobacillus abundance was not significantly associated with any of the bone density measurements in the univariate and bivariate models unless BMI was included in the model and therefore was no longer considered;

11：g_Bacteroides is negative correlation with FN BMD and hip T-score.

**Table S7 Taxonomic Diversity (continued)**

|  | **Enrich in OP** | **Enrich in HC** | **No difference between group** |
| --- | --- | --- | --- |
| **(Wang et al. 2017a)** | p_Chlamydiae, p_Nitrospirae, p_Acidobacteriota,, f_Comamonadaceae, f_Caldilineaceae, f_Anaerolineaceae, f_Gemmatimonadaceae |  |  |
| **(Lv et al. 2021)** | c_Bacilli | c_Clostridia, o_Clostridiales, f_Verrucomicrobiaceae |  |
| **(Rettedal et al. 2021)** | Bacteroides_Bacteroides_uniformis, g_Adlercreutzia，c_Betaproteobacteria | d_Archaea, o_Methanobacteriales, p_Euryarchaeota, f_Methanobacteriaceae, c_Methanobacteria, g_Romboutsia, g_Turicibacter， f_Peptostreptococcaceae |  |
| **(He et al. 2020)** |  | Bacteroides massiliensis, Lachnospira pectinoschiza, Bacteroides coprocola |  |
| **(Di et al. 2021)** |  |  | f_Bifidobacteriaceae^, g_Butyricicoccus^, g_Bifidobacterium^, p_Actinobacteria^, p-Proteobacteria^, g_Gemmiger^ |
| **(Wei et al. 2021)** | g_Eisenbergiella*#, g_Flavonifractor# | g_Raoultella# | g_Coprococcus`* |
| **(Wang et al. 2017b)** |  |  | p_Proteobacteria，p_Actinobacteria，g_Prevotella, g_Lachnoclostridium, g_Pseudobutyrivibrio, g_Klebsiella, g_Megamonas, g_Bifidobacterium, Escherichia-Shigella, g_Enterococcus, g_Streptococcus, g_Phascolarctobacterium, g_Anaerostipes, g_Roseburia |
| **(Das et al. 2019)** | g_Actinomyces |  | p_Actinobacteria, p_Proteobacteria, p_Verrucomicrobia, g_Roseburia, g_Coprococcus, g_Ruminococcus, g_Prevotella, g_Clostridium_XI, g_Anaerostipes, g_Bifidobacterium, g_Streptococcus, g_Akkermansia, g_Alistipes, g_Lachnospira, g_Clostridium_IV, g_Clostridium_sensu_stricto, Escherichia/Shigella, Dorea |
| **(Qin et al. 2021)** | Actinomyces graevenitzii, Actinomyces sodontolyticus, Olsenella unclassified, Pantoea unclassified, Streptococcus gordonii, Streptococcus mitis oralis pneumoniae, Streptococcus parasanguinis, Streptococcus sanguinis, and pathogenic bacteria, such as Escherichia coli | Akkermansia muciniphila, Bacteroides eggerthii, Bacteroides fragilis, Bacteroides uniformis, and g_ Paraprevotella |  |
| **(Xu et al. 2020)** | o_Bacteroidales, f_Prevotellaceae | o_Erysipelotrichales, f_Erysipelotrichaceae | g_Hungatella, g_Agathobacter, g_Zymomonas, g_Megamonas, g_Citrobacter, p_Proteobacteria, p_Actinobacteria, p_Cyanobacteria， p_Fusobacteria, p_Melainabacteria, p_Euryarchaeota, p_Tenericutes, p_Verrucomicrobia |

Notes: OP: Osteoporosis group; HC: Health control group; ^Before regression, enrich in HC; `Before regression, enrich in OP;* Found in absolute abundance; # Found in relative abundance.

**Search Strategy**

1. **PUBMED**

**Result: 229**

**Term1:** Bone Density

#1: **((((((("Bone Density"[Mesh]) OR (Bone Densities[Title/Abstract])) OR (Density, Bone[Title/Abstract])) OR (Bone Mineral Density[Title/Abstract])) OR (Bone Mineral Densities[Title/Abstract])) OR (Density, Bone Mineral[Title/Abstract])) OR (Bone Mineral Content[Title/Abstract])) OR (Bone Mineral Contents[Title/Abstract])**

Term2: Osteoporosis

#2: **"osteoporosis, postmenopausal"[MeSH Terms] OR "osteoporosis"[MeSH Terms]**

Term3: Gastrointestinal Microbiome

#3: **(((((((((((((((((((((((((((((((((((((Gastrointestinal Microbiome[MeSH Terms]) OR (Gastrointestinal Microbiomes[Title/Abstract])) OR (Microbiome, Gastrointestinal[Title/Abstract])) OR (Gut Microbiome[Title/Abstract])) OR (Gut Microbiomes[Title/Abstract])) OR (Microbiome, Gut[Title/Abstract])) OR (Gut Microflora[Title/Abstract])) OR (Microflora, Gut[Title/Abstract])) OR (Gut Microbiota[Title/Abstract])) OR (Gut Microbiotas[Title/Abstract])) OR (Microbiota, Gut[Title/Abstract])) OR (Gastrointestinal Flora[Title/Abstract])) OR (Flora, Gastrointestinal[Title/Abstract])) OR (Gut Flora[Title/Abstract])) OR (Flora, Gut[Title/Abstract])) OR (Gastrointestinal Microbiota[Title/Abstract])) OR (Gastrointestinal Microbiotas[Title/Abstract])) OR (Microbiota, Gastrointestinal[Title/Abstract])) OR (Gastrointestinal Microbial Community[Title/Abstract])) OR (Gastrointestinal Microbial Communities[Title/Abstract])) OR (Microbial Community, Gastrointestinal[Title/Abstract])) OR (Gastrointestinal Microflora[Title/Abstract])) OR (Microflora, Gastrointestinal[Title/Abstract])) OR (Gastric Microbiome[Title/Abstract])) OR (Gastric Microbiomes[Title/Abstract])) OR (Microbiome, Gastric[Title/Abstract])) OR (Intestinal Microbiome[Title/Abstract])) OR (Intestinal Microbiomes[Title/Abstract])) OR (Microbiome, Intestinal[Title/Abstract])) OR (Intestinal Microbiota[Title/Abstract])) OR (Intestinal Microbiotas[Title/Abstract])) OR (Microbiota, Intestinal[Title/Abstract])) OR (Intestinal Microflora[Title/Abstract])) OR (Microflora, Intestinal[Title/Abstract])) OR (Intestinal Flora[Title/Abstract])) OR (Flora, Intestinal[Title/Abstract])) OR (Enteric Bacteria[Title/Abstract])) OR (Bacteria, Enteric[Title/Abstract])**

**Final search strategy: (#1 OR #2) AND #3**

1. COCHRANE

RESULT: 2

Search strategy:

**#1: MeSH descriptor: [Bone Density] explode all trees**

**#2: MeSH descriptor: [Osteoporosis] explode all trees**

**#3: #1 OR #2**

**#4: MeSH descriptor: [Gastrointestinal Microbiome] explode all trees**

**#5: #3 AND #4**

1. WoS

RESULT: 679

Search strategy:

**#1: (((((((TS=(Bone Density)) OR TS=((Bone Densities)) OR TS=(Density, Bone)) OR TS=(Bone Mineral Density)) OR TS=(Bone Mineral Densities)) OR TS=(Density, Bone Mineral)) OR TS=(Bone Mineral Content)) OR TS=(Bone Mineral Contents))**

**#2: (((TI=(osteoporosis, postmenopausal)) OR TI=(osteoporosis, postmenopausal)) OR TI=(osteoporosis)) OR TI=(Osteoporoses)**

**#3: #1 OR #2**

**#4: (((((((((((((((((((((((((((((((((((((TS=(Gastrointestinal Microbiome)) OR TS=(Gastrointestinal Microbiomes)) OR TS=(Microbiome, Gastrointestinal)) OR TS=(Gut Microbiome)) OR TS=(Gut Microbiomes)) OR TS=(Microbiome, Gut)) OR TS=(Gut Microflora)) OR TS=(Microflora, Gut)) OR TS=(Gut Microbiota)) OR TS=(Gut Microbiotas)) OR TS=(Microbiota, Gut)) OR TS=(Gastrointestinal Flora)) OR TS=(Flora, Gastrointestinal)) OR TS=(Gut Flora)) OR TS=(Flora, Gut)) OR TS=(Gastrointestinal Microbiota)) OR TS=(Gastrointestinal Microbiotas)) OR TS=(Microbiota, Gastrointestinal)) OR TS=(Gastrointestinal Microbial Community)) OR TS=(Gastrointestinal Microbial Communities)) OR TS=(Microbial Community, Gastrointestinal)) OR TS=(Gastrointestinal Microflora)) OR TS=(Microflora, Gastrointestinal)) OR TS=(Gastric Microbiome)) OR TS=(Gastric Microbiomes)) OR TS=(Microbiome, Gastric)) OR TS=(Intestinal Microbiome)) OR TS=(Intestinal Microbiomes)) OR TS=(Microbiome, Intestinal)) OR TS=(Intestinal Microbiota)) OR TS=(Intestinal Microbiotas)) OR TS=(Microbiota, Intestinal)) OR TS=(Intestinal Microflora)) OR TS=(Microflora, Intestinal)) OR TS=(Intestinal Flora)) OR TS=(Flora, Intestinal)) OR TS=(Enteric Bacteria)) OR TS=(Bacteria, Enteric)**

**#5: #3 AND #4**

1. EMBASE

RESULT: 499

Search strategy:

**#1: ‘intestine flora’/exp**

**#2: ‘osteoporosis’/exp**

**#3: ‘bone density’/exp**

**#4: #2 OR #3**

**#5: #1 AND #4**

1. Chinese database

RESULT: CNKI:187

VIP:58

WanFang:55

SinoMed:32

Search strategy:

骨密度OR骨量AND肠道菌群
